# Are pressures sores in nursing homes related to nursing home size? A cross-sectional analysis

**DOI:** 10.1093/eurpub/ckac131.559

**Published:** 2022-10-25

**Authors:** N McGrane, P Dunbar, A Luding, L Keyes

**Affiliations:** 1 LENS Projects, Health and Information Quality Authority of Ireland, Dublin, Ireland; 2 School of Public Health, University College Cork, Cork, Ireland

## Abstract

**Background:**

Incidence of pressure sores are an indicator of quality of care provided. Pressure sores requiring hospitalisation are a very poor outcome and may indicate a chronic unmet need. Organisational factors of services are modifiable factors and may be associated with occurrence and severity of pressure sores. However, little investigation has been completed on this to date. The aim of this study was to assess the association of size of nursing homes and pressure sores requiring hospitalisation.

**Methods:**

Using the Database of Statutory Notifications from Social Care in Ireland we identified from notifications received by the regulator in 2019, the number of pressure sores that required hospitalisation from nursing homes. Association of size of service (registered beds) and the percentage of pressure sores requiring hospital treatment was investigated, using negative binomial regression, unadjusted and adjusted for provider type (company, sole trader, statutory body, unincorporated body, partnership), staff to resident ratio and distance to hospital (km), and nurse to resident ratio as a proxy for resident complexity in a further model.

**Results:**

598 Nursing homes were in operation in 2019. 1 was excluded for missing covariate data and 597 included for analysis. 447 submitted 2996 notifications of pressure sores, median (IQR): 3 (0 to 8). 255 pressure sores (8.51% of total) required hospitalisation. Pressure sores that required hospitalisation and size of service was positively associated, incidence rate ratio (95% confidence interval): 1.001 (1.000 to 1.002). This remained when adjusted for covariates, 1.003 (1.001 to 1.006) and additionally for nurse to staff ratio, 1.013 (1.011 to 1.016)

**Conclusions:**

A small but significant higher number of pressures sores required hospitalisation from larger nursing homes. The reasons for this are unknown but may include cultural differences and ability to provide continuity of care between larger and smaller nursing homes.

**Key messages:**

• Larger nursing homes are associated with higher incidents of pressure sores that require hospitalisation. This association remains when adjusted for covariates and nurse to staff ratio.

• Reasons larger nursing homes are associated with higher incidents of pressure sores that require hospitalisation are unknown but may be influenced by their ability to provide continuity of care.

